# Mediators of Inflammation: Inflammation in Cancer, Chronic Diseases, and Wound Healing

**DOI:** 10.1155/2015/570653

**Published:** 2015-10-15

**Authors:** Caigan Du, Madhav Bhatia, Sydney C. W. Tang, Mingzhi Zhang, Theodore Steiner

**Affiliations:** ^1^Department of Urologic Sciences, The University of British Columbia, Jack Bell Research Centre, Vancouver, BC, Canada V6H 3Z6; ^2^Department of Pathology, University of Otago, Christchurch 8140, New Zealand; ^3^Department of Medicine, The University of Hong Kong, Queen Mary Hospital, Pokfulam, Hong Kong; ^4^Department of Medicine, Vanderbilt Center for Kidney Disease, Vanderbilt University, Nashville, TN 37232, USA; ^5^Department of Medicine, The University of British Columbia, Jack Bell Research Centre, Vancouver, BC, Canada V6H 3Z6

Chronic diseases and conditions, such as kidney and liver failure, cancers, and diabetes, are the leading causes of morbidity and mortality, and, according to the data published by the World Health Organization [1], these diseases caused 38 million deaths in 2009 alone, more than 62% of all deaths around the world. However, the pathogenesis of these diseases is not fully understood yet. In more recent years, evidence indicates the link of a form of low-degree systemic and chronic inflammation to many types of chronic diseases including cancer [2–6], suggesting the inflammation as a common risk factor for these diseases.

What is inflammation? The word* inflammation* comes from the Latin “*inflammo*,” meaning “*blaze, burn*,” and is defined in biology as “*the body's immune system's response to stimulus*” by the US National Library of Medicine. The inflammation is commonly ignited by the pathogen (bacteria, viruses, or fungi) infection, tissue injuries and remodeling, or nonphysiological cell death, and is typically viewed as a self-protective response and is important for wound healing. However, in most cases, prolonged or chronic inflammation leads to the pathological changes in body tissues or organs, an inflammatory disease. To understand how the inflammation contributes to the chronic diseases including cancer, we have collected eight clinical observation and experimental studies in this special issue focusing on the mediators of inflammation in chronic diseases and cancer. Using systemic review and meta-analysis, W. Wang et al. have shown an association of gout with an increased risk of cancer, particularly urological cancers, digestive system cancers, and lung cancer. Similarly, there is a positive correlation of serum uric acid levels with total cancer incidence, but it is only found in males not in females (S. Yan et al.). Gout is an inflammatory response to the accumulated urate crystals in the joint that may be formed due to the high levels of uric acid in the blood, in which uric acid crystals have been identified as an endogenous stimulus for activation of the immune responses, particularly interleukin-1*β*-mediated inflammation via activation of the NOD-like receptor protein 3 (NLRP3) inflammasome [7]. How this inflammatory response is related to tumor development and why this association is gender-dependent are not known. The association of a panel of inflammation mediators, such as interleukin-8 and tumor necrosis factor- (TNF-) *α*, with chronic diseases (liver disease and gastritis) and different types of cancer (liver and gastric cancer and melanoma) has been documented in this issue (T. Liu et al.; A. Essadik et al.; C.-D. Ene et al.). Whether upregulation of these mediators is part of pathogenesis of these diseases or is just an associated risk factor requires further investigation. Interestingly, T. Liu et al. summarize that c-Myc, a protooncogene, may play a central role in the development of liver inflammation as well as liver cancer. Is mutation of c-Myc required for the stimulation of inflammation in the liver? A similar question is for the study by A. Essadik et al.; whether the mutation at TNF-*α*−238 (G/A) allele found in patients with gastric pathologies promotes the transcription of TNF-*α* or that at TNF-*α*−193 (G/A) downregulates its expression needs further investigation. Finally, we do need well-designed experimental studies to answer our question, the role of inflammation in chronic diseases, carefully. G. C. W. Chan et al. present a good example for that by showing different effects from others in literature of N-acetyl-seryl-aspartyl-lysyl-proline (Ac-SDKP) or Captopril treatment on the attenuation of interstitial injury or macrophage in a mouse model of chronic kidney disease. In summary, chronic diseases including cancer somehow affect everyone's life, and we do not know the exact cause of these diseases and how to effectively treat or prevent them as of today. Numerous studies including the papers published in this issue clearly show the association of inflammation with the development of chronic diseases, but we have more questions about the role of inflammation in the pathogenesis of these diseases than the answers we have to that as of today.

*Caigan Du* *Caigan Du* *Madhav Bhatia* *Madhav Bhatia* *Sydney C. W. Tang* *Sydney C. W. Tang* *Mingzhi Zhang* *Mingzhi Zhang* *Theodore Steiner* *Theodore Steiner*
